# Metformin Decreases 2-HG Production through the MYC-PHGDH Pathway in Suppressing Breast Cancer Cell Proliferation

**DOI:** 10.3390/metabo11080480

**Published:** 2021-07-26

**Authors:** Sehyun Oh, Youngup Cho, Minsun Chang, Sunghyouk Park, Hyuknam Kwon

**Affiliations:** 1 Natural Product Research Institute, College of Pharmacy, Seoul National University, 1 Gwanak-ro, Gwanak-gu, Seoul 08826, Korea; ohsean367@gmail.com; 2 Department of Surgery, College of Medicine, Inha University, Inhang-Ro 27, Chung-gu, Incheon 22332, Korea; yucho@inha.ac.kr; 3 Department of Biological Sciences, College of Science, Sookmyung Women’s University, 100, Cheongpa-ro 47-gil, Yongsan-gu, Seoul 140-742, Korea; 4 Department of Biological and Environmental Sciences, University of Helsinki, 00160 Helsinki, Finland

**Keywords:** metformin, metabolomics, 2-HG, PHGDH, anticancer effect

## Abstract

The biguanide drug metformin has been widely used for the treatment of type 2 diabetes, and there is evidence supporting the anticancer effect of metformin despite some controversy. Here, we report the growth inhibitory activity of metformin in the breast cancer (MCF-7) cells, both in vitro and in vivo, and the associated metabolic changes. In particular, a decrease in a well-known oncometabolite 2-hydroxyglutarate (2-HG) was discovered by a metabolomics approach. The decrease in 2-HG by metformin was accompanied by the reduction in histone methylation, consistent with the known tumorigenic mechanism of 2-HG. The relevance of 2-HG inhibition in breast cancer was also supported by a higher level of 2-HG in human breast cancer tissues. Genetic knockdown of PHGDH identified the PHGDH pathway as the producer of 2-HG in the MCF-7 cells that do not carry isocitrate dehydrogenase 1 and 2 (IDH1/IDH2) mutations, the conventional producer of 2-HG. We also showed that metformin’s inhibitory effect on the PHGDH-2HG axis may occur through the regulation of the AMPK-MYC pathway. Overall, our results provide an explanation for the coherent pathway from complex I inhibition to epigenetic changes for metformin’s anticancer effect.

## 1. Introduction

Metformin belongs to the biguanide-class drugs and has been used for decades for the treatment of type 2 diabetes [1,2]. Its excellent safety profile, including a low incidence of hypoglycemia, and low cost have made it the most frequently prescribed antidiabetic drug in modern days [2,3] when more target-oriented antidiabetic drugs are available [4]. At the systemic level, metformin inhibits hepatic gluconeogenesis and glucose output [5] and increases insulin sensitivity in the muscles [6]. The most proximal cellular target of metformin is thought to be mitochondrial complex I, but the downstream effects seem to be more complex [7,8]. The molecular mechanism of its antidiabetic effects has long been thought to involve the activation of AMP-activated protein kinase (AMPK) [9], but other studies, especially those involving animals lacking AMPK, strongly suggest the presence of alternative mechanisms [10,11]. These may have to do with a decrease in cyclic AMP (cAMP) synthesis, and the resulting inhibition of protein kinase A (PKA), a key enzyme in glucagon signaling for the regulation of glycolysis and gluconeogenesis [11]. A retrospective study also reported reduced cancer incidences among diabetic patients treated with metformin [12]. This initial result was followed by other studies supporting the beneficial effect of metformin for colorectal and breast cancer patients with diabetes [13,14], although contradicting results have also been reported [15]. The effect of metformin on cancers has been suggested to be mediated either indirectly through insulin or directly through mammalian target of rapamycin (mTOR) modulation [7]. The indirect insulin-dependent mechanism is based on insulin’s growth-promoting effect on tumor cells [16], and the direct insulin-independent mechanism may occur both through AMPK dependently [17] or independently through Rag-GTPase responses to energy status [18]. Still, the mechanistic details and the effect of metformin on non-diabetic cancer patients are subjects of further studies. In addition, the exact cancer patient population that will benefit most from the metformin treatment is not well known. Many pre-clinical research and clinical trials are underway to address these unknown aspects of metformin’s effect on cancer [19,20,21]. Phosphoglycerate dehydrogenase (PHGDH) catalyzes the thermodynamically reversible conversion between 3-phosphohydroxypyruvate and 3-phosphoglycerate (3PG) [22]. This reaction is the first step in serine biosynthesis from the glycolytic intermediate, 3PG. Several studies have also implicated PHGDH in tumorigenesis in some cancers, such as breast cancer and melanoma [23,24]. For breast cancer, PHGDH has been shown to be overexpressed mostly in estrogen receptor-negative subtypes, including triple-negative subtypes. Still, serine synthesis by PHGDH itself may not fully account for the tumorigenic roles of PHGDH, given that serine supplementation to the PHGDH-knockdown cells did not rescue their growth inhibition [23]. PHGDH was also shown to generate an oncometabolite 2-hydroxyglutarate (2-HG) in PHGDH-amplified triple-negative breast cancer cells [25], suggesting that this non-canonical activity of the enzyme may contribute to its tumorigenic role.

The oncogenic role of 2-HG was first suggested when a mutant isocitrate dehydrogenase (IDH MT) in brain cancers was shown to produce it as a result of the neomorphic activity [26]. The 2-HG has been shown to competitively inhibit alpha-ketoglutarate-dependent dioxygenases including histone demethylases and TET methylcytosine hydroxylases [27,28]. These inhibitions can cause a perturbation in the methylation status of the nucleosome, which may contribute to the tumorigenic processes [29]. Although the 2-HG production has been connected to the tumorigenesis by IDH mutations, several studies also showed IDH mutation-independent production in leukemia and breast cancer samples [30,31]. Along with the above-mentioned 2-HG generation by PHGDH, these results suggest that the inhibition of 2-HG production may be of therapeutic value in relevant cancers with an IDH wildtype (WT) and a high level of 2-HG. Here, using metabolomic profiling followed by mechanistic verification, we show that metformin suppresses the growth of the MCF-7 estrogen receptor-positive breast cancer cell line through the inhibition of 2-HG production by PHGDH. Our results may prove a new mechanism for metformin’s anticancer effect and help identify metformin-susceptible tumors.

## 2. Results

### 2.1. Xenograft Study on Metformin’s Cancer Prevention Effect

**Figure 1:**
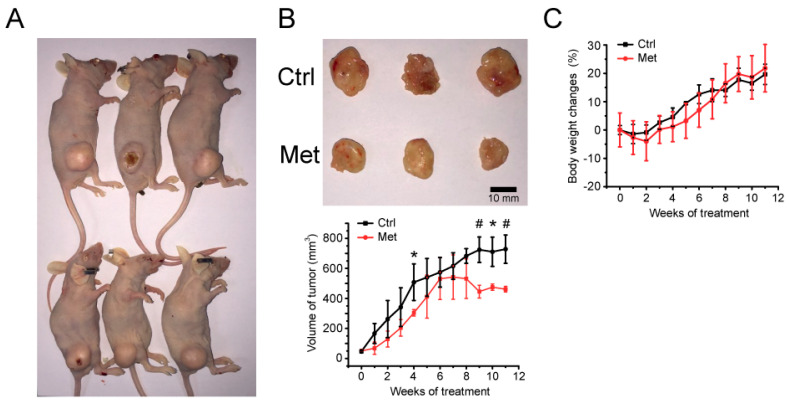
Mouse xenograft model of MCF-7 cells and metformin’s relevance in vivo. (**A**) MCF-7 cells were implanted subcutaneously into the right flank of BALB/c nude mice. Upper: control; lower: metformin treated. (**B**) The effect of 300 mg/Kg metformin daily administration on the growth of MCF-7 xenograft tumors. The upper panel shows sample tumors from 11 weeks post-metformin administration. The tumor size was measured once a week, and the average tumor tissue volume is represented in the lower panel with the mean ± standard deviations (n = 3). (**C**) Mouse body weight changes were determined every week after metformin administration. *, # statistically significant difference in tumor size. *: *p* < 0.05, #: *p* < 0.01.

To investigate the relevance of metformin’s effect against breast cancer cells, we established an in vivo xenograft mouse model bearing MCF-7 cells (Figure 1A). After the oral administration of metformin to xenograft mouse, metformin-fed mice exhibited significantly lower tumor volumes (Figure 1B) compared to the control group, with little difference in body weights (Figure 1C). 

Metformin was administrated daily at a 300 mg/Kg dose in drinking water. The blood concentration of metformin could be about 24 to 31 mM, assuming the mouse total blood volume to be 58.5 to 75 mL/Kg. This concentration is within the range of the metformin concentration used in other studies [32,33,34,35]. The clinical dose for the metformin administration is between 1500 mg/day to 2000 mg/day which roughly corresponds to 1.6 mM to 2.2 mM. Despite the relatively high dose of metformin administration here, no serious problem with the change in mouse body weight was observed, which demonstrates the relevance of the concentration used in our experiments.

### 2.2. Metformin’s Inhibitory Activity against Breast Cancer Cells

**Figure 2:**
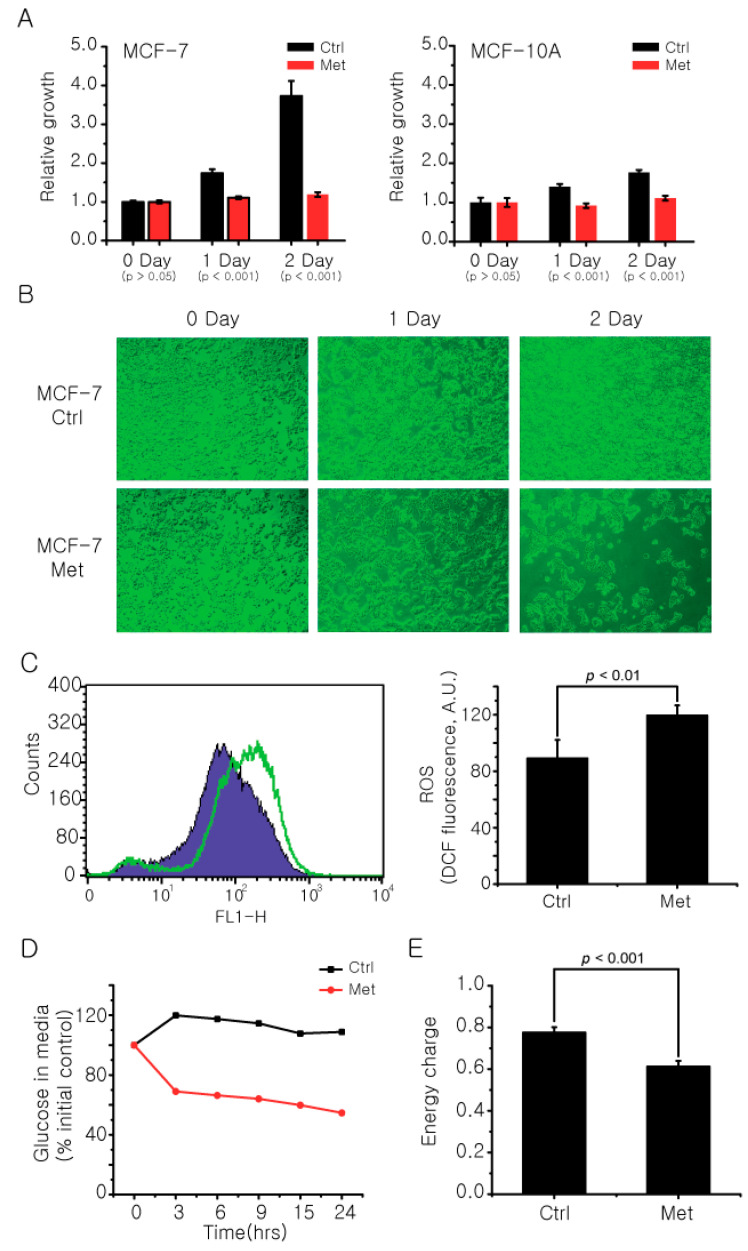
Metformin affects cancer cell growth and metabolic phenotypes. The final concentration of 20 mM metformin was treated to MCF-7 and MCF-10A cells for 2 days and the cell viability at each day was measured by a standard MTT assay (**A**) and microscope (**B**). (**C**) The change in ROS levels in MCF-7 cells after metformin treatment was measured using a fluorescent dye DCFH-DA. (**D**) The difference in glucose uptake by metformin treatment was compared by measuring glucose level in MCF-7 cell culture media using an NMR spectrometer. The relative amounts were normalized against the 0 h point. (**E**) The change in energy charge of MCF-7 cells after metformin treatment was calculated with absolute concentrations of AMP, ADP, and ATP measured with LC/MS using the following equation: ([ATP] + 0.5 [ADP])/([ATP] + [ADP] + [AMP]). Ctrl: control group; Met: Metformin treated group. The data are from three independent experiments. The error bars represent the standard deviation.

There have been debates on metformin’s effectiveness in breast cancer treatment and prevention. To observe whether metformin exhibits inhibitory activity against breast cancer cells, we treated metformin to the MCF-7 ER-positive cancer cell line with MCF-10A as a benign control. The MTT assay showed that metformin significantly inhibited the growth of the MCF-7 cells with a much smaller effect on the MCF-10A cells (Figure 2A), and the same inhibitory effect was observed under a microscope (Figure 2B). The growth inhibition of MCF-7 cells by metformin was accompanied by other phenotypic changes such as changes in reactive oxygen species (Figure 2C) and glucose uptake (Figure 2D), and a decrease in ATP energy charge in the cell (Figure 2E). These results suggest that metformin has a differential inhibitory effect on MCF-7 cells, compared to normal cells, with metabolic implications.

### 2.3. Metformin Induces Metabolomic Changes and Decreases 2-HG Level in MCF-7 Cells

We next carried out metabolomics studies to characterize the metabolic effects of metformin on the inhibition of MCF-7 cell growth. NMR and LC/MS metabolomic data were obtained from MCF-7 and MCF-10A cells with and without metformin treatment. We identified the metabolites detected by the two methods, performed univariate analysis, and listed significant ones in Table 1. 

**Figure 3:**
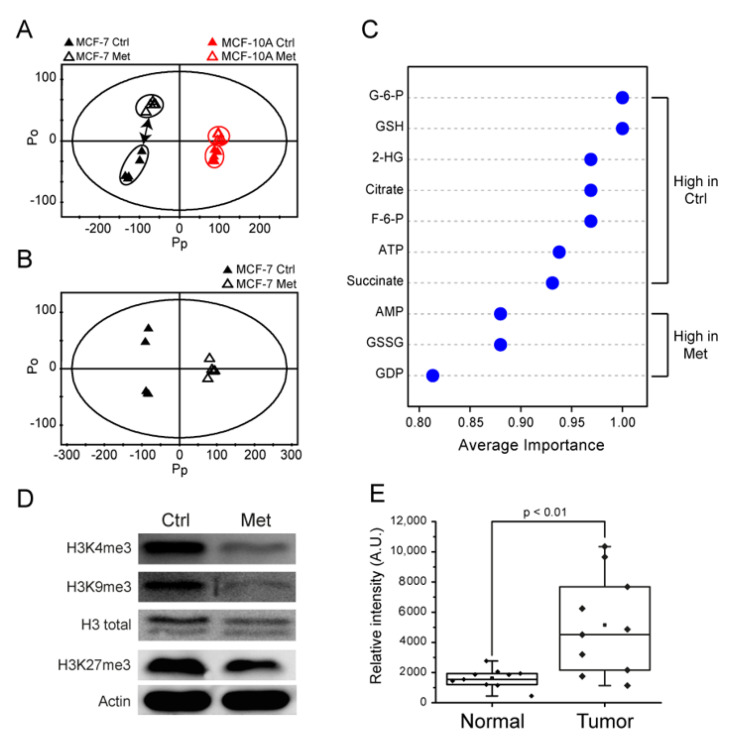
Multivariate analysis on metabolomic changes, histone methylation status by metformin treatment in MCF-7 cells, and the 2-HG level in breast cancer tissues from patients. (**A**,**B**) NMR metabolomic changes by metformin treatment were analyzed by multivariate analysis. Black symbols: MCF-7; red symbols: MCF-10A; filled symbols: Control (Ctrl) group; open symbols: Metformin (Met) treated group. (**A**) Principal component analysis (PCA) on metformin-treated and control groups for MCF10a and MCF7 cells. (**B**) Orthogonal projections to latent structure-discriminant analysis (OPLS-DA) for control and metformin-treated MCF-7 cells. (**C**) Multiple ROC curve analysis for the significance of contributing metabolites using the quantitative LC/MS analysis. (**D**) The changes in the histone methylation status of MCF-7 cells were measured by Western blot using H3K4me3, H3K9me3, and H3K27me3 antibodies after metformin treatment. The data are representative of three independent experiments. (**E**) The 2-HG levels from paired normal (non-involved) and tumor tissues from breast cancer patients were measured by LC/MS (n = 10 for each group).

We further analyzed the NMR metabolomic data holistically using multivariate analysis. Principal component analysis (PCA) showed that the metformin treatment shifted the overall metabolomic profiles of both MCF-7 and MCF-10A cells (Figure 3A), but the change was more pronounced in MCF-7 cells. This was consistent with the larger effect of metformin on the growth of MCF-7 than MCF-10A cells. Then, the orthogonal projections to latent structure-discriminant analysis (OPLS-DA) was performed on MCF-7 cells to investigate the metabolomic changes that contribute to the growth inhibition by metformin treatment. Figure 3B shows that metformin treatment exhibited clearly distinguishable metabolomic changes that may be attributable to several metabolites (Figure 3C). The metformin-treated MCF-7 cells exhibited higher levels of AMP, GDP, and GSSG and lower levels of glycolytic-TCA metabolites (G-6-P, F-6-P, and ATP; citrate and succinate) and 2-HG (Figure 3C). Of these, 2-HG was among the highest-ranking contributor, and its ~70% decrease upon metformin treatment caught our attention, as it is an established oncometabolite that exists in high levels in a variety of cancers including glioma and AML [36]. 

### 2.4. Metformin Changes Histone Methylation Status of MCF-7 Cells

With the link between metformin treatment and 2-HG modulation, we tested whether the metformin-induced decrease in the 2-HG level causes changes to the epigenetic methylation status. It has been known that the oncogenic mechanism of 2-HG generated by IDH mutations involves the inhibition of alpha-ketoglutarate-dependent dioxygenases [29], ultimately leading to the epigenetic methylation changes [27,37]. Therefore, we measured histone methylation using anti-H3K4me3, H3K9me3, and H3K27me3 antibodies, and the results showed reduced histone methylation in MCF-7 cells treated with metformin (Figure 3D). Consequently, this increased histone demethylation by metformin seems to be consistent with the decreased histone demethylation observed in IDH MT cells with high a 2-HG level [37]. On the other hand, we could not observe significant changes in the DNA methylation status (Supplementary Figure S1). Overall, our results suggest that histone epigenetic changes may be involved in the growth inhibitory effects of metformin through a 2-HG reduction in MCF-7 cells.

### 2.5. The 2-HG Level Is Higher in Tumor Tissue Samples

Then, we tested the relevance of 2-HG and its reduction by metformin observed in breast cancer cells with clinical samples. We measured the 2-HG level in the breast cancer and the adjacent non-involved tissues (n = 10 for each group). The LC/MS analysis showed that the 2-HG level in the cancer tissues was significantly higher than that in the normal tissues (Figure 3E). Although this is a retrospective pilot result, it suggests that 2-HG production may be involved in breast cancer and a target for a possible therapy. A larger-scale test for the 2-HG level and the relevance of metformin treatment in a prospective cohort are warranted. 

### 2.6. Metformin Reduces 2-HG Level through Downregulation of PHGDH

**Figure 4:**
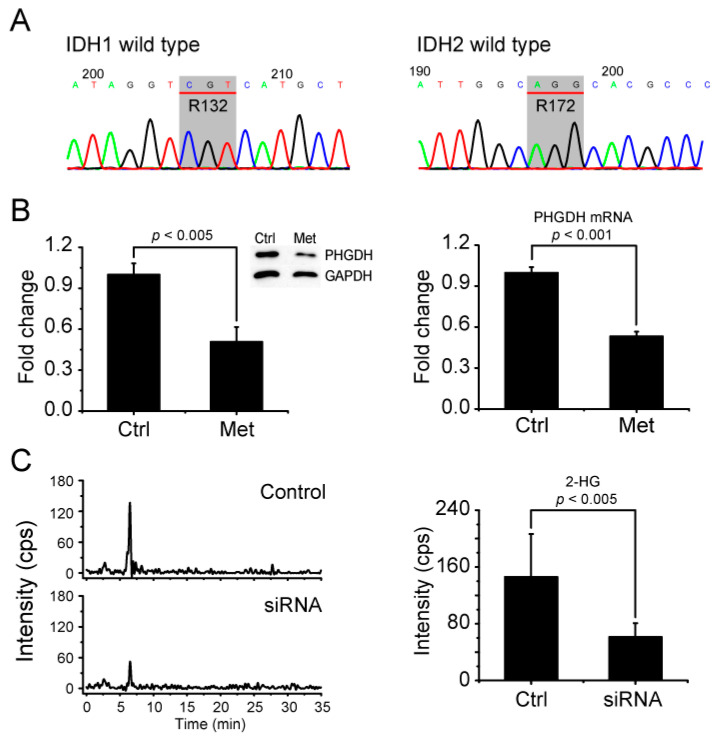
Metformin inhibits 2-HG production by PHGDH in MCF-7 cells without IDH mutations. (**A**) DNA sequencing against IDH1 R132 and IDH2 R172 residues was performed using genomic DNA from MCF-7 cells. Codons for IDH1 R132 and IDH2 R172 are indicated in gray boxes as CGT and AGG encoding Arginine. (**B**) The PHGDH level after metformin treatment was measured for protein (left) and mRNA (right) by Western blot and real-time PCR, respectively. (**C**) The alteration of the 2-HG level by PHGDH siRNA was measured with LC/MS. The data are from five independent experiments. The error bars represent the standard deviation.

Next, we investigated how 2-HG is produced in MCF-7 cells. Many studies have shown that 2-HG is highly elevated in glioma and AML cells harboring IDH mutations. However, one study showed that about 40% of AML patients with a high 2-HG level have an IDH WT genotype [30]. Therefore, we carried out DNA sequencing for IDH1 and IDH2 genes in MCF-7 cells, both of which turned out to be wildtype (Figure 4A). The results suggest that 2-HG is not produced by IDH MT in MCF-7 cells and that its reduction by metformin treatment does not arise from the inhibition of IDH MT. There was a previous study suggesting that PHGDH can also produce 2-HG in triple-negative breast cancer cells [25]. Therefore, we tested for the possibility that 2-HG reduction by metformin in MCF-7 cells may occur through the drug’s effects on PHGDH. Western blot and real-time PCR showed that both the PHGDH protein and mRNA levels are significantly reduced by metformin treatment in MCF-7 cells (Figure 4B). To further prove that the reduction of 2-HG by metformin treatment occurs through its modulation of PHGDH, we knocked down PHGDH with siRNA and measured the 2-HG level using LC/MS. The siRNA treatment lowered the PHGDH level (Supplementary Figure S2) and concomitantly inhibited the 2-HG generation (Figure 4C). These results strongly suggest that metformin’s effect on 2-HG occurs through its downregulation of PHGDH in MCF-7 cells. 

### 2.7. AMPK-MYC Pathway Is Implicated in the Regulation of PHGDH-2HG by Metformin

**Figure 5:**
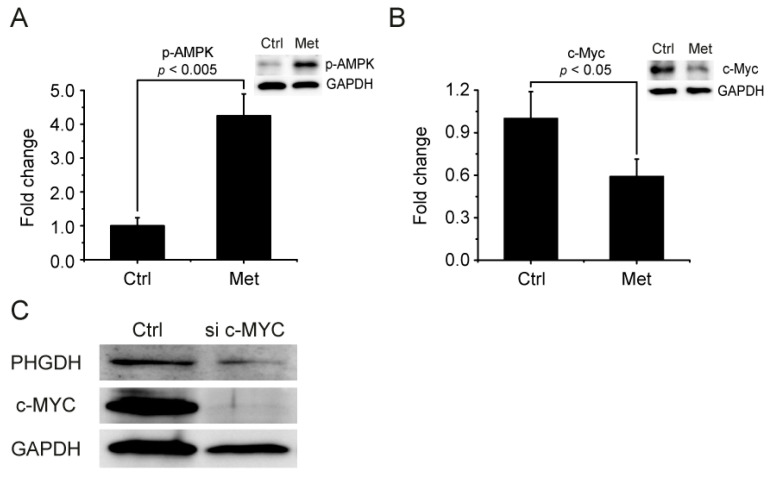
Metformin inhibits PHGDH through the AMPK-MYC pathway. (**A**) Changes in (**A**) p-AMPK and (**B**) c-MYC levels by metformin treatment were measured by Western blot. (**C**) The effect of c-MYC siRNA on PHGDH was estimated by Western blot.

Possible upstream mediators for metformin’s regulation of the PHGDH-2HG pathway were also investigated. As previous studies revealed that PHGDH can be regulated by MYC [38,39] and that MYC is, in turn, down-regulated by AMPK activation [40,41], we tested the involvement of the AMPK-MYC pathway. Metformin treatment significantly activated AMPK, as judged by the phosphorylation of the enzyme (Figure 5A). At the same time, the MYC level was also significantly decreased by metformin (Figure 5B), consistent with the previous report that MYC is degraded by activated AMPK [40,41]. We also knocked down MYC with siRNA and observed a decreased PHGDH level, which established the link between MYC and PHGDH (Figure 5C). These results implicate the AMPK-MYC pathway in the regulation of the PHGDH-2HG axis by metformin.

## 3. Discussion

The effect of metformin on cancers is multifaceted, and may not be explained by single-target effects [42]. At the patient level, many clinical studies are underway to determine whether metformin is effective for particular cancer prevention or treatment [20,43]. At the molecular level, multiple mechanisms have been proposed for its antitumor effect, such as the insulin-dependent, AMPK-dependent, or AMPK-independent pathways [7,44]. These not only reflect the complex nature of the effect of metformin, but may also suggest the inherent heterogeneity of different cancer tissues or even cancer cell lines. Therefore, in our pursuit of discovering the metabolic effect of metformin on breast cancers at a molecular level, we focused on a single ER-positive breast cancer cell line, MCF-7, and tested the extrapolations of the results reported for the triple-negative breast cancers. The significant inhibition of the MCF-7 xenograft tumor by metformin in vivo confirms the relevance of our experimental system. Our results suggest that metformin inhibits the growth of MCF-7 cells, which seems to occur through metformin’s inhibitory effect on 2-HG production. We expanded PHGDH’s roles from producing 2-HG in triple-negative breast cancer cells [25], to the regulation of metformin’s growth inhibitory effect on ER-positive breast cancer cells. The effect of metformin on the histone methylation status is consistent with this suggestion. Interestingly, a related result was recently reported, showing an increased toxic effect of metformin on MCF-10A engineered to over-produce 2-HG by mutant IDH1 R132H [45]. Overall, our study suggests a possible mechanistic linkage among metformin’s effect on cancer cells [46], PHGDH’s roles [25], and 2-HG production in ER-positive breast cancer cells [31]. This is also consistent with the elevated 2-HG level in breast carcinoma cell lines and its possible usage as a predictive marker for malignancy [47], and PHGDH’s roles in tumor formation and breast cancer metastasis [48]. The most plausible upstream pathway for the modulation of the PHGDH-2HG link by metformin appears to be metformin’s inhibition of c-Myc through activated AMPK [40,41]. It is well established that metformin activates AMPK by increasing the AMP level (low energy charge) through its inhibition of mitochondrial complex I, and it is one of the important upstream mechanisms for metformin’s antidiabetic and antitumor effect [7]. Consistently, we observed an increase in AMP and a decrease in ATP by metformin treatment. In addition, we showed AMPK activation by metformin treatment. A recent study reported that MYC induces over-production of 2-HG and DNA epigenetic changes, which were related to a poor prognosis in a subset of breast cancers [31]. In addition, glutaminase was overexpressed in those tumors, which may contribute to 2-HG generation by supplying the carbon source. Other studies also suggested that MYC can upregulate PHGDH [38]. Taken together, our results showing the link between metformin and the PHGDH–2HG axis can suggest a coherent mechanistic flow from complex I inhibition to epigenetic changes for the growth suppression of the ER-positive MCF-7 cells by metformin. Although the 2-HG production has been connected to the tumorigenesis by IDH mutations, recent studies also showed 2-HG production to be independent of IDH mutations in leukemia and breast cancer samples [49,50]. Along with the above-mentioned 2-HG generation by PHGDH, our results suggest that the inhibition of 2-HG production may be a therapeutic strategy for relevant breast cancers with an IDH WT and a high level of 2-HG. 

It should be noted that not all previous results are in line with the proposed mechanistic aspects of metformin’s anticancer effect. First, we observed changes in the histone methylation status upon metformin treatment and the following 2-HG inhibition, but could not observe concomitant DNA methylation changes. In comparison, DNA hypermethylation was observed, but the histone methylation status was not clear for 2-HG over-producing breast cancer tumors [31]. In addition, leukemia cells from AML patients with IDH1/2 mutations exhibited DNA hypermethylation patterns at global and specific gene levels [51]. Still, it has been reported that histone methylation can induce cancer-related epigenetic silencing independent of DNA methylation [52]. Furthermore, histone hypermethylation was observed in IDH MT-transfected adipocytes with defective differentiation, but DNA hypermethylation was absent in promotors for adipogenesis and differentiation [37]. The same study also found that increased histone trimethylation was observed from IDH MT glioma patient samples compared to IDH WT ones. Therefore, the histone and DNA methylation status in 2-HG over-producing cancers could be either enzyme (IDH, PHGDH, or else) or cancer tissue type (triple-negative vs. ER-positive or leukemia vs. glioma or else)-dependent. Secondly, our result of PHGDH downregulation by metformin was obtained using the MCF-7 cells, an ER-positive cell line without PHGDH gene amplification or overexpression. In comparison, PHGDH overexpression has been reported mainly in ER-negative, especially, triple-negative breast cancer cells [23,24]. In addition, 2-HG over-production by PHGDH was first demonstrated in triple-negative breast cancer cells [25]. Tests involving a wider range of cells and tumor types may prove the relevance of the ER status in metformin’s effects. 

Although metformin has been used extensively for diabetes, its effect can vary among patients. Initially, an organic cation transporter 1 (OCT1) genetic status was proposed to affect the antidiabetic responses [53], but later studies found that other transporters are also important [54,55]. For metformin’s effects on cancers, many reports suggest reduced cancer risk or survival benefits for diabetic breast or colon cancers [12,13,14,15,56,57,58]. However, others pointed out biases in study designs [15] or an absence of the association between metformin and mortality in a population-based study [59]. These uncertainties may be related to the lack of understanding of the detailed mechanisms of metformin’s effects and its targets. In this respect, a recent report is worth noting, where metformin exhibited survival benefits only in diabetic breast cancer patients with an ER/PR-positive and HER2-positive status [60]. Therefore, defining a potential patient sub-population that can best benefit from metformin may require further refinement. As we showed that metformin’s anticancer effect is related to 2-HG inhibition by the downregulation of PHGDH and c-MYC in ER-positive cells, a future clinical study may reveal whether the 2-HG and/or PHGDH level can serve as a criterion for patient categorization for metformin’s response.

## 4. Materials and Methods

### 4.1. Reagents

Metformin (1,1-Dimethylbiguanide hydrochloride, D150959, Sigma), 2′,7′-Dichlorofluorescin diacetate (DCF-DA, D6883, Sigma), β-Estradiol (E4389, Sigma), DMSO (D8418, Sigma), thiazolyl blue tetrazolium bromide (M2128, Sigma), cholera toxin (C8052, Sigma), EGF (E9644, Sigma), hydrocortisone (H0135, Sigma) and insulin solution (I0516, Sigma) were obtained from Sigma-Aldrich (St. Louis, MO, USA). Metformin, EGF, and hydrocortisone were dissolved in distilled water to make high concentration stock. Oligofectamine (12252011) was purchased from Invitrogen (Waltham, MA, USA) and Matrigel Matrix (354248, Corning, MA, USA) was obtained from the local distributor. Pierce™ ECL Western Blotting Substrate (32109, Thermo Scientific, Waltham, MA, USA) and ImageQuant LAS4000 (GE Healthcare Life Sciences, Pittsburgh, PA, USA) were used for western blot analysis. DMEM (11995-074, Gibco), DMEM/F12 (11330-032, Gibco), FBS (16000-044, Gibco), Trypsin-EDTA (25200-056, Gibco), penicillin streptomycin (15240-062, Gibco), and MEM non-essential amino acids (11090-081, Gibco) were purchased from Gibco (Grand Island, NY, USA). Antibodies for western blot were purchased from Cell Signaling technology (Danvers, MA, USA), Santa Cruz Biotechnology (Santa Cruz, CA, USA), Abcam (Cambridge, MA, USA), and Millipore (Billerica, MA, USA); PHGDH antibody (13428, Cell Signaling Technology), GAPDH antibody (sc-31915, Santa Cruz Biotechnology), β actin (sc-47778, Santa Cruz Biotechnology), H3 total antibody (9715, Cell Signaling Technology), H3K4me3 Antibody (A2357, Abcam), H3K9me3 Antibody (A2360, Abcam), H3K27me3 Antibody (EPR18607, Abcam), phosphor AMPK Antibody (ab133448, Abcam), c-MYC Antibody (ab86356, Abcam). 

### 4.2. Cell lines and Metformin Treatment

Breast cancer cell line MCF-7 and benign breast epithelial cell line MCF-10A were from ATCC (Manassas, VA, USA). For routine maintenance of cells, MCF-7 and MCF-10A cell lines were cultured in their respective media (MCF-7: DMEM including 10% FBS, 1% MEM non-essential amino acids, and 1% penicillin streptomycin; MCF-10A: DMEM/F12 including final 10% FBS, 100 ng/mL cholera toxin, 20 ng/mL EGF, 500 ng/mL hydrocortisone, and 10 µg/mL Insulin, and 1% penicillin streptomycin). For actual experiments, MCF-7 cells grown in its own media were put through a media adaptation process for 24 h in the MCF-10A media to make the environments the same for both MCF-10A and MCF-7 cells. Metformin was treated after the media adaptation at the final concentration of 20 mmol/L metformin for 24 h. Cell viability after metformin treatment was measured by the standard colorimetric MTT reduction assay. The MCF-7 cells were sequenced for the mutations of IDH1 and IDH2 genes at Macrogen Inc. (Seoul, Korea). 

### 4.3. Xenograft Mouse Model with MCF-7 Cells

Five-week-old female BALB/c nude mice (supplied from CLS Bio, Bucheon, Korea) were used for the in vivo experiment. All animal housing and experiments were performed under approved protocols from Seoul National University Institutional Animal Care and Use Committees (approved number; SNU-160721-1-1). The mice were acclimatized for one week before the beginning of the experiment. A mixture of 100 µL matrigel (Corning, NY, USA) and 100 µL DPBS containing 1.5 × 10^7^ MCF-7 cells was injected subcutaneously on the right flank of the mice. With the injection of the cells, mice were fed 200 µg/mL β-Estradiol (water soluble, Sigma-Aldrich, MO, USA) in drinking water for six weeks to facilitate the MCF-7 tumor growth [61]. Two weeks after the cell injection, mice were divided into two groups and only one group was treated with metformin (300 mg/Kg body weight daily in drinking water) by oral administration. The concentration of metformin was calculated based on the water intake of the animals suggested from the Johns Hopkins University Animal Care and Use Committee (1.5 mL/10 g body weight/day). Tumor size was measured weekly with a caliper and calculated as follows: tumor size (mm^3^) = [length (mm) × width (mm) × height (mm)] π/6.

### 4.4. Metabolomic Analysis and Metabolite Measurements

NMR and LC/MS metabolomics analysis, sample preparation, data acquisition, and data processing were performed as described previously [49,50]. Energy charge was calculated from the AMP, ADP, and ATP concentration measured with LC-MS using the following equation: ([ATP] + 0.5 [ADP])/([ATP] + [ADP] + [AMP]). Glucose uptake was assessed by measuring the glucose concentration in the media using 1D proton NMR [62]. The culture media was mixed with 4 times volume of methanol-acetonitrile mixture (5:3). The sample was then centrifuged at 15,000 rpm for 20 min at 4 degrees and the supernatant was dried using a vacuum concentrator. Dried samples were resuspended with 500 µL of the NMR sample buffer (2 mM Na_2_HPO_4_ and 5 mM NaH_2_PO_4_ in D_2_O with 0.025% TSP (trimethylsilylpropionic acid sodium salt-d4). The re-dissolved samples were transferred to 5 mm NMR tubes and the glucose peak at 5.22 ppm on 1D proton NMR spectra was integrated. At each time point, the glucose peak area was normalized against the 0 h point.

### 4.5. ROS Measurement

We detected the ROS changes using the fluorescence probes-based method (DCF-DA method) which have been widely used by conjugation with the flow cytometry [63]. Cells in 1 mL PBS were incubated with 3 µM of 2′-7′-Dichlorofluorescein diacetate (DCF-DA) in light-protected microtubes for 30 min at 37 degrees and washed with PBS. Then, the cells were resuspended with 1 mL of PBS and analyzed with the FACS Calibur^®^ flow cytometer with CellQuest Pro software (BD Biosciences, San Jose, CA, USA).

### 4.6. Quantitative Real-Time PCR and Western Blot Analysis

Total RNA extraction and cDNA synthesis were conducted with the Easy-Spin total RNA extraction kit (iNtRON Biotechnology Inc., Seongnam, Korea) and the High-Capacity cDNA Reverse Transcription Kit (Applied Biosystems, Foster City, CA, USA) following the manufacturer’s protocols. Primers for PHGDH and GAPDH (Bioneer, Daejeon, Korea) were synthesized with the following sequences: PHGDH sense 5′-AACTTCTTCCGCTCCCATTT–3′, PHGDH antisense 5′–GTCATCAACGCAGCTGAGAA–3′, GAPDH sense 5′–GAGTCAACGGATTTGGTCGT–3′, GAPDH antisense 5′–TTGATTTTGGAGGGATCTCG–3′. For the DNA amplification, iTAQ™ SYBR Green Supermix with ROX (Bio-Rad, Hercules, CA, USA) and the synthesized cDNA were used according to the manufacturer’s instructions on the Applied Biosystem’s 7500 fast system. Western blot experiments were performed using routine procedures. 

### 4.7. 2-HG Quantification from Human Breast Tissue Samples 

We obtained 10 paired sets of human normal breast tissues and breast cancer tissues from the Inha University Hospital Biobank (IUHB) at Incheon, Korea, and the Biobank of Pusan National University Hospital at Pusan, Korea. Tissue samples were stored at -80 degrees until use. Frozen tissue samples were transferred into 1.5 mL microtubes containing 400 µL of methanol–chloroform solvent mixture (2:1) at 4 degrees. The samples were homogenized with a pellet pestle motor (Kimble Chase^®^, Vineland, NJ, USA) on ice, and 200 µL of chloroform and 200 µL of distilled water were added. The homogenized samples were vortexed for 30 sec and centrifuged at 15,000 rpm for 30 min at 4 degrees, and the 350 µL supernatant was dried with a vacuum concentrator. Dried samples were resuspended and applied to an LC/MS system for 2-HG level measurement. Pellets located in the middle phase after centrifugation were resuspended with 1 mL of 1 M urea buffer, and the total protein concentrations were measured by a BCA assay for normalization.

### 4.8. DNA and Histone Methylation Analysis 

For the DNA methylation analysis, total genomic DNA was extracted from harvested cells using the G-DEXTM IIc Genomic DNA Extraction Kit (iNtRON Biotechnology Inc., Seongnam, Korea). The methylation status was measured using the MethylFlash^TM^ methylated DNA quantification kit (P-1035-48, EPIGENTEK, New York, NY, USA) and MethylFlash^TM^ hydroxymethylated DNA quantification kit (P-1036-48, EPIGENTEK, New York, NY, USA) following the manufacturer’s instructions. Histone methylation was measured using Western blot analysis with an antibody against trimethylated H3K4, H3K9, and H3K27. 

### 4.9. PHGDH Knockdown with siRNA

For the siRNA treatment, 2 × 10^5^ MCF-7 cells were seeded in a 6-well cell culture plate, and 200 nmol/L of siRNA (Bioneer, Daejeon, Korea) and oligofectamine (Invitrogen, Grand Island, NY, USA) mixture were added. After 24 h of culture, the media adaptation process was carried out as mentioned above, followed by another 24 h-incubation with fresh MCF-10A culture medium. The PHGDH siRNA (sense 5′-UCUAACCUUGGAGCUCACU(dTdT)-3′; antisense 5′-AGUGAGCUCCAAGGUUAGA(dTdT)-3′, Bioneer, Daejeon, Korea) and the negative control siRNA (Bioneer, Daejeon, Korea) were used in the experiment.

## Figures and Tables

**Table 1:** Metabolite changes in MCF-7 cells after metformin treatment measured by an NMR and LC/MS spectrometer.

	Metabolites	Fold Change (%)	*p* Value
Increased	Acetate	43.54	<0.001
	Alanine	18.65	<0.001
	AMP	46.17	<0.005^3^
	Creatine	196.27	<0.001
	GDP	38.50	<0.01
	Glutamine	87.94	<0.001
	GSSG	153.39	<0.001
	Isoleucine	78.43	<0.001
	Leucine	67.51	<0.001
Decreased	ATP	47.72	<0.05
	Citrate	55.23	<0.001
	F-6-P	66.49	<0.001
	GSH	17.52	<0.005
	G-6-P	69.91	<0.001
	Lactate	19.37	<0.05
	O-Phosphocholine	33.08	<0.001
	Succinate	84.07	<0.05
	2-HG	68.85	<0.005

## Data Availability

All datasets generated and/or analyzed during the current study are available in the manuscript or from the corresponding author upon reasonable request (approved number; SNU-160721-1-1).

## Supplementary Materials

The following are available online at https://www.mdpi.com/article/10.3390/metabo11080480/s1, Figure S1: DNA methylation status following metformin treatment in MCF-7 cells, Figure S2: PHGDH knockdown by siRNA treatment.
