# Supplementary material for: Metformin Decreases 2-HG Production through the MYC-PHGDH Pathway in Suppressing Breast Cancer Cell Proliferation
Source: Metabolites. 2021 Jul 26;11(8):480. doi: 10.3390/metabo11080480 (PMC8402004; doi:10.3390/metabo11080480)
Supplement: Supplementary file 1 [file metabolites-11-00480-s001.zip › metabolites-1283650-SI.pdf]

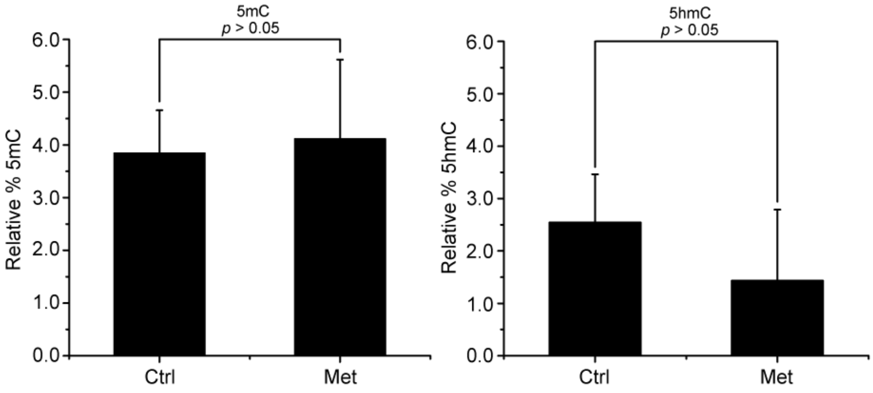

Supplementary Figure S1. DNA methylation status following metformin treatment in MCF-7 cells

DNA methylation was measured for total genomic DNA with MethylFlash™ methylated DNA Quantification Kit and MethylFlash™ Hydroxymethylated DNA Quantification Kit (EPIGENTEK, New York, NY, USA) following manufacturer's instructions. The data are from three independent experiments. The error bars represent standard deviation.

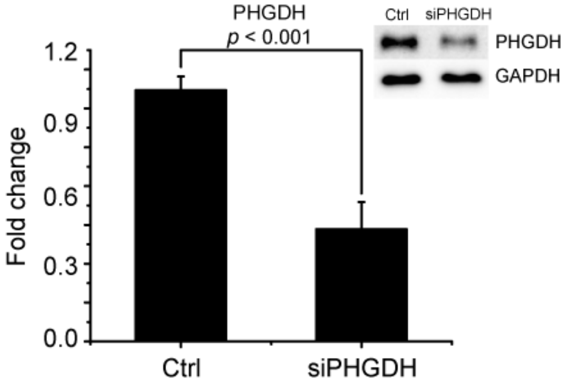

Supplementary Figure S2. PHGDH knockdown by siRNA

The PHGDH fold change was estimated by western blot analysis (upper right inset). The data are from five independent experiments. The error bars represent standard deviation.
